# Perspectiva histórica de la importancia de la agremiación odontológica

**DOI:** 10.21142/2523-2754-1004-2022-127

**Published:** 2023-12-26

**Authors:** Anton Samplonius Angobaldo, Jaime Otero-Injoque

**Affiliations:** 1 Facultad de Estomatología, Universidad Nacional Mayor de San Marcos. Lima, Perú. a_samplonius@hotmail.com Universidad Nacional Mayor de San Marcos Facultad de Estomatología Universidad Nacional Mayor de San Marcos Lima Peru a_samplonius@hotmail.com; 2 Carrera de Estomatología, Universidad Científica del Sur. Lima, Perú. joteroi@cientifica.edu.pe Universidad Científica del Sur Carrera de Estomatología Universidad Científica del Sur Lima Peru joteroi@cientifica.edu.pe

La presente editorial brinda una reseña histórica respecto de la asociación gremial como uno de los elementos más relevantes de la práctica odontológica moderna y fundamenta la importancia de los colegios odontológicos contemporáneos en el desarrollo y el avance de la profesión dental. El término gremio se define como un conjunto de personas que tienen el mismo oficio o profesión, y que se asocian para ordenarlo, defenderlo y compartir experiencias. Este vocablo involucra a la totalidad de individuos que trabajan en una misma rama profesional, lo cual contrasta con el concepto de sindicato, que implica una reunión voluntaria de un grupo de estos. 

Los gremios han existido casi desde el inicio de la historia humana, cuando la comunidad productora comenzó a especializarse en tareas sociales de cierta especificidad, como los pastores, tejedores, alfareros, agricultores, sacerdotes, médicos, etc. En las antiguas civilizaciones del mundo, se evidenció la costumbre de que los practicantes de un mismo oficio formaran corporaciones para reglamentar y ordenar su ejercicio, proteger la dignidad y evitar el abuso tanto de contratantes como de contratados. Por ejemplo, existe registro de este tipo de asociación entre los constructores de las pirámides de Egipto y en la antigua Roma. En la España medieval, el primer gremio registrado fue el de los albañiles, en el siglo XIII.

En el medioevo europeo, dichas corporaciones fueron denominadas gremios y se organizaron contractualmente con base en el binomio maestro y aprendiz, con lo que jugaron un papel importante durante la etapa precapitalista. Incluso, estuvieron prohibidas o normadas desde que se conformó el capitalismo, por considerarse contrarias a las libertades necesarias para el mercado. Actualmente, a nivel mundial, se cuenta con los colegios profesionales como una figura fundamental dentro de las corporaciones gremiales.

En la etapa precolombina del Perú antiguo no hay evidencia científica considerable sobre la realidad histórica de los gremios y solo se cuenta con unos pocos datos de la época colonial. Por ejemplo, en el campo de los antecesores del actual cirujano dentista, Ricardo Palma^1^ narra en su tradición “La gran querella de los barberos” que, en 1626, había 29 barberos en Lima. Cuenta que la Iglesia pretendió impedirles ejercer los domingos, por motivos religiosos, aunque ese día existía una gran demanda de sus servicios; por ello, a pesar de la amenaza de excomunión, los barberos plantearon y ganaron un “recurso de fuerza”.

En su Memorial de la Historia del Nuevo Mundo, de 1631, fray Buenaventura de Salinas y Córdova^2^ contabilizó doce boticas, nueve médicos, diez cirujanos y cincuenta y tres tiendas de barberos y cirujanos españoles en la Lima colonial. Narra el fraile que, hacía unos años, se había fundado el gremio de barberos y sacamuelas, con la advocación de Santa Apolonia. Como cuenta su contemporáneo Juan Antonio Suardo^3^, en 1637, los barberos y cirujanos salían en procesión el Viernes Santo de la iglesia de Nuestra Señora de la Soledad, en la plaza San Francisco del centro histórico de Lima. Señala, además, que eran abanderados, en la romería de ese año, por Juan de Vega, junto a otros barberos y cirujanos. Dicha narración coincide con lo señalado por Ricardo Palma, como una evidencia que detona el inicio del gremio odontológico en el Perú.

Se dispone de dos informantes convergentes que confirman la posible primera agremiación de barberos sacamuelas de Lima, sucedida a comienzos del siglo XXVII. Este antecedente histórico de la agremiación profesional en odontología invocaba el mismo espíritu que insufla el marco normativo del actual Colegio Odontológico del Perú: reglamentar, ordenar y proteger a su agremiado.

Desde fines del siglo XIX, en la época republicana, el dentista desplazó al barbero y al flebótomo como el encargado de la terapéutica dental. Sin embargo, no contó con la confianza de la sociedad. Técnica y socialmente, el oficio de dentista estaba relegado a pobres niveles de estima y reconocimiento. Se le consideraba una labor mecánica y manual, que solo requería experiencia, cierta habilidad manual o psicomotricidad y don de palabra. En este sentido, cabe destacar que el término “palangana”, que es un lavabo de latón como el que usaba Don Quijote en su cabeza, refiere a un instrumento propio del oficio del barbero sacamuelas y, a la vez, se vincula con los términos fanfarrón y parlanchín.

Las plazas públicas y los mercados eran el espacio en el que los dentistas de la época laboraban, como lo narra con maestría José Gálvez Barrenechea^4^ en su libro Una Lima que se va. Son descritos como “trajeados llamativamente, llenos de garambainas y guirindolas”, y por esa razón se les consideraba individuos pintorescos y algo charlatanes.

No se tienen muchos datos desde la independencia nacional hasta finales del siglo XIX sobre la evolución de la comunidad de dentistas. Sin embargo, todo indica que en 1891 se fundó la Asociación Dental de Lima con la participación de nombres como Juan Jenkins, Christian Dam, Francisco Gaillour, Walter Stubs y David Castro. Dicha agrupación es el primer intento moderno y conocido de juntar esfuerzos por el bien común de todos los profesionales de los dientes. De esta institución matriz surgieron la Asociación Dental del Perú, fundada en 1914 por Ernesto Febres Odriozola, y el Círculo Odontológico de 1919, liderado por Alberto Galliani. En 1920, se estableció también la Sociedad Odontológica del Perú. 

Todas estas instituciones fueron efímeras en duración y recordación, lo que concuerda con el espíritu integracionista de la época. En 1922 se estableció la Federación Odontológica, con Ernesto Dam y Pedro Ayllón a la cabeza. De allí, podemos dar un salto a la reconocida Academia de Estomatología del Perú, fundada en 1929 con la presidencia de Ricardo Salazar Southwell y la primera vicepresidencia de Federico Schuetz. Dicha entidad era considerada la “Casa del Odontólogo”^5^.

La Asociación Odontológica del Perú nació en 1945, para transformarse en 1961 en el sindicalista Colegio Odontológico Funcional, con Mariano Flores Rodríguez a la cabeza. Se tiene referencia que en dicha época también se fundaron agrupaciones en torno a las distintas especialidades odontológicas^6^.

Durante la Segunda Convención Odontológica Nacional de 1960, se determinó el imperativo de fundar un colegio odontológico con un reglamento apropiado a las circunstancias históricas y la ebullición política y gremial de entonces. Dicho anhelo se plasmó en la Ley 15251, promulgada el 4 de diciembre de 1964 por el entonces presidente de la república Fernando Belaunde Terry y el Congreso de la República. Así, nace el Colegio Odontológico del Perú con su primer decano, Óscar Serrudo Vásquez de Peralta^7^.

Las funciones de la institución rectora de la odontología peruana van evolucionando con el tiempo e involucran progresivamente a más actores y partes influyentes e interesadas^8^. El reto de integrar a los dentistas entre sí se complementa con la necesidad de articular a los agremiados con los pacientes, las entidades reguladoras y otros actores sociales. De acuerdo con el devenir histórico, ahora las funciones del colegio profesional son más específicas y complejas que antes. Abarcan el ordenamiento del ejercicio profesional mediante un código deontológico basado en la teoría ética moderna; la representación y la defensa de los intereses de los diferentes miembros del gremio ante posibles abusos o imposiciones de otros actores o poderes públicos; el resguardo de los derechos que amparan a los pacientes y consumidores del servicio odontológico; la contribución con tareas de formación profesional en aquellos campos no cubiertos por la universidad y la academia; el ofrecimiento a los colegiados de servicios de bienestar como seguros de salud, bibliotecas, bolsa de empleos, etc.; y la generación de una identidad grupal mediante actividades culturales, exposiciones y museos, para compartir y perennizar la experiencia que nos une.
